# Monthly Summary

**Published:** 1874-10

**Authors:** 


### MONTHLY SUMMARY.

---

*School Diseases.*—It is a serious question whether we are not getting what is called education at too exorbitant a price, when the health and usefulness of the eyes are impaired or sacrificed. And the mischief that is done to eyes in schools and colleges may safely be taken as an indication of the damage that is inflicted upon other parts of the body. Objectors may, perhaps, say that the appalling statistics obtained by the foreign observers could not be gathered in American schools and colleges. I believe that they might, and I found my belief upon twenty years' work among just the classes of subjects tabulated by Cohn and other Continental observers. I believe that our system of education, if, indeed, we may be said to have a system, is one of the most damaging in its effects upon the growing bodies of scholars of any in the world. Let any one familiar with hygiene take the pains, as I have, to enquire carefully into the physical effects of curricula of our leading schools and colleges, and he will be compelled to confess that there is the greatest cause for reform. The attention which is paid to gymnastic exercises and other methods of physical culture does not correct the evils. It often happens that those who really need physical exercise most do not get it, or that the exercise is excessive, and does harm to those who engage in it. What we need in our school and college curricula is a diminution of the hours of labor. The working hours too often extend from eight or nine in the morning to ten or eleven at night. The strain thus put upon growing bodies is too great. Some method should be devised by which much that now involves a persistent use of the eyes in confined and unnatural postures of the body could be accomplished through the use of models or photographs, or the blackboard. Much that is now attempted to be taught by badly-printed books might be taught orally or by some form of object lessons. Even if such radical changes could not be accomplished, much might be done towards lessening the evil effects of our present method by shortening the hours devoted to study, by correcting defects in the architecture of class and study rooms, by improving the ventilation, heating, and lighting of school-houses, and by diffusing information among the parents of scholars, so that there may be less in the home-life that is prejudicial to health. And just here we touch the very fountain of the evil. Our schools cannot be much, if any, above the intelligence of their patrons. I do not blame the teachers for the evils in our systems of education. I blame boards of trustees and other school and college boards for not applying the principles that have already been worked out by scientific men. If architects and boards of managers of schools and colleges would apply in the construction and conduct of their institutions of learning even a few of the principles that sanitarians all agree upon, we would at once see a reduction in those forms of disease which are traceable to their present neglect.—*C. R. Agnew, M. D., Sanitarian.*

---

*Camphorated Phenol.*—In a note on this subject *Campania Medica and Gazetta Medica Italiana-Lombardia*, November 8, after noticing the chemical and therapeutic properties of carbolic acid, BUFALINI goes on to speak of its behaviour when combined with camphor.

In making experiments with carbolic acid for the purpose of preserving animal substance from putrefaction, Bufalini met with a peculiar phenomenon when it was in contact with camphor. When about equal parts of carbolic acid and camphor are dissolved in alcohol, in about twelve or thirteen hours there arises to the surface of the solution a yellowish stratum of oily appearance; it does not mix with the liquid or water, nor is the camphor contained in the alcohol precipitated by water. All this indicates that a chemical combination has taken place, forming a substance which Bufalini calls camphorated phenol.

In preparing this compound, Bufalini prefers the two following methods. In the first, one part of carbolic acid and two of camphor broken into small pieces are mixed in a vessel and allowed to stand for some hours, when a reddish yellow oily liquid will be formed; this is camphorated phenol, which is purified by washing with cold water. The second method consists in dissolving three parts of carbolic acid in ten of alcohol, and five of camphor in twelve of alcohol, mixing the solution in a wide-mouthed vessel, and allowing the mixture to stand for a day or two; the camphorated phenol rises to the top, and may be removed by simple decantation.

Prepared in either of these ways, camphorated phenol is a liquid of oily appearance, reddish yellow or wine-red in colour, having a smell of camphor, insoluble in water, but soluble in alcohol and ether.

Regarding its therapeutic uses, the author gives the following as his conclusions.

1. Camphorated phenol produces the same effects as carbolic acid, but is less dangerous. It may be used both externally and internally—*e. g.* in enteric fever and other infectious disorders.

2. It has the power of modifying unhealthy wounds, and of destroying the parasites which are present in certain diseases, as septicæmia, typhoid forms of fever, etc.

3. The medical use of camphorated phenol is to be preferred to that of carbolic acid, as the former does not present the disadvantages of the latter.

4. Camphorated phenol, when applied to the wounds, does not irritate them, or act as a caustic, or disorganizing substance on them; and may be used in large doses, without producing symptoms of poisoning.—*London Med. Record.*

---

*Anæsthetization During Sleep.*—In reply to the oft-asked question, "Can a person be anæsthetized during sleep?" Dr. W. R. Cluness reports (*Pacific Med. and Surg. Journ.*, June, 1874) two cases of successful chloroformization during sleep.

The first case was that of a girl aged eight years, in whom, as a sequel to acute otitis media, the mastoid cells of one side became inflamed. Dr. E. M. Curtis deemed it expedient to operate for the evacuation of the pus, and met me the following morning for this purpose. On our arrival we learned that our patient had slept but little during the night, but was then sleeping sweetly. Chloroform was at once administered upon a four-by-six piece of surgeon's lint, held as near the child's mouth as possible without coming in actual contact. Not the slightest effort was made by the child to avoid the inhalation of the anæsthetic, and in a few moments she was well under its influence, and the operation was performed and the wound dressed before she had fully gained consciousness. On making my evening visit, I was informed that my patient was not aware that she had undergone a severe surgical operation.

My second case occurred in the person of a little girl two and a half years old, brought to me for the purpose of having a supernumerary toe removed from each of its feet. While waiting for the arrival of Dr. Nelson, who assisted in the operation, the child fell asleep and was placed in the operating chair. As soon as the doctor arrived, chloroform was administered in the manner already detailed in the former case, and with equal success.

In the first case the condition of the child probably favoured the ready induction of anæsthesia, while in the second, age alone could be supposed to have influenced the result.—*Med. News and Libary.*

---

*Influence of Fear in the Production of Disease.*—Dr. O. Kohls. (*Berlin Klin. Wochenschr., Chicago Jour. Nervous and Mental Diseases*) records the following observations made by himself during the bombardment of Strasburg. A great variety of diseases were evidently either produced or greatly aggravated by sudden fright, from various causes, during the siege. Among affections of the central nervous system, he reports three cases of paralysis agitans, two women and one man, and three cases of spinal paralysis. He also saw affections of the genital system, suppression of the menses and abortions, and one case of angina pectoris, following the shock of sudden fright in a healthy person. Diseases of the respiratory apparatus were notably aggravated. The first appearance of hæmoptisis was often dated to sudden terror from the events of bombardment. Three cases of icterus catarrhalis were traced to the same cause. One case of affection of joints is given. The patient, hitherto a sound man, was, by the explosion of a shell near by, rendered speechless and trembling for several hours. He immediately noticed a painful swelling of the hand and knee joints, with stiffness of the right index finger, which lasted for a considerable period.—*Detroit Review of Medicine and Pharmacy.*

---

*The Position of Woman.*—In all the situations and pursuits of life the Almighty has established bounds or limitations beyond which woman cannot go without defeating the primary objects of her creation. The reasons are obvious. It gradually changes her organization. By a physiological law of supply and demand, nature, in the case of woman, makes certain drafts monthly upon her constitution. That this law of periodicity be properly observed is indispensable for good health and the highest development both of the body and mind. Again, if the brain is reflectively exercised too much, the body suffers; so of the brain alone, there cannot be steady strain upon certain portions of it without impairing the functions of other parts. Maternity is primary law in her creation. Physiology, pathology, records of health. disease and mortality, establish the fact that this is her NORMAL state. In the observance of this law, certain physical conditions are indispensable; there must be a proper development of those portions of the body concerned in this function; neither can they answer the demands nature makes if kept constantly impoverished.—*Nathan Allen, M. D., LL D., Sanitarian.*

---

*The Hours of Maximum Mortality in Acute and Chronic Diseases.*—Dr. James Finlayson classifies the hours of death in a large number of well observed cases—both acute and chronic. In the latter he shows the greatest mortality to take place between the hours of eight and twelve A. M. In acute cases, the maximum mortality occurred between four and eight A. M. The next greatest was between four and eight P. M. The importance of these observations is evident. If it is desirable for a patient to live the greatest length of time, then especial vigilance will be observed during the above critical periods in using every means for recruiting the vital energies.—*Glasgow Med. Jour.*

---

*Chlorate of Potash in Cancer.*—Dr. von Burow, Sen. (*Berl. Klin. Wochenschrift*) has noticed that energetic local use of chlorate of potash would cause a shrinkage in ulcerating cancers; that resorption of neighboring infiltration took place; that the secretion was lessened, the sensitiveness reduced. A case of caries of the lower jaw, with extensive infiltration, and large crater-like swelling, was soon healed by the same treatment, the chlorate of potash powdered, and sprinkled on the ulcerations —*Rundschau.*

---

*The Champion Chloroform Tippler.*—Dr. W. W, Parker related, at the last meeting of the Virginia State Medical Society, the case of a man named Johnson, a blacksmith, who became addicted to chloroform tippling How much in quantity he swallowed is not stated, but he bought and drank three thousand dollars worth in three years! His mind then became affected and he imagined himself tricked. Meanwhile he fattened fifty pounds, and on ceasing the habit lived fifteen years in good health, and died from natural causes.

